# Heavy about His Embonpoint

**Published:** 1910-06

**Authors:** 


### Heavy About His Embonpoint.

Henry Beach Needham, who met Colonel Roosevelt when he landed in Europe, describes the colonel as being the very picture of health and in the pink of condition except that he still has about twenty pounds of superfluous flesh. He says:

"Before Roosevelt left the White House the 'twenty pounds' was nearer forty. Despite his violent exercise—including the hundred-mile ride—he was beginning to show heaviness about his jaws and embonpoint. All that is gone now. He seems not to have a pound of superfluous weight. His eyes are markedly bright—and nothing gets by them. His skin is clear and his cheeks hard. Wrinkles are scarcely to be found—indeed, many a woman on the 'right side of forty' would be delighted if her face were as free from crow's feet."

A careless reading of this description of the great naturalist is calculated to alarm the average reader. That "heaviness about the jaw and embonpoint" is likely to mislead those of our readers who speak only United States, and even that with a strong Texas accent, but there is no danger in "embonpoint." It is not a disease, indeed, it is not even embonpoint, when spoken, but "arnbompwar," the last syllable to be blown out as it were with the mouth wide open and the lips flapping like a barn door in a high wind. When spoken this way it is entirely harmless and not contagious. Not claiming to be authority on this subject, for in truth we would not know an embonpoint if met right in the middle of the road. We are glad to learn from careful investigation that Colonel Roosevelt is in no danger from this particular embonpoint or any number of embonpoints, whether blown at him with mouth wide open or hissed at him in sibilant whispers through set teeth. He has met more deadly and more dangerous foes than embonpoint, and Mr. Needham's statement that he was "heavy about the jaws and embonpoint" only evidences the continual vigorous growth of cheek that will always keep pace with his embonpwar.—*Austin Statesman.*

---

Stuart H. Boman, of West Virginia, a member of the board of trustees of the Peabody College for Teachers, is authority for the statement that it is more than probable that Peabody will be located adjacent to and affiliated with Vanderbilt University, and that a $10,000,000 endowment will be made by Eastern capitalists. He further states that he believes these same men will put up money to launch the greatest medical school in America by a consolidation of all the medical colleges in Nashville.—*Woman's National Daily,* March 10.

